# Causes of hospitalization and mortality in persons with epilepsy: The EpiLink Bologna cohort, Italy

**DOI:** 10.1111/ene.16576

**Published:** 2025-01-31

**Authors:** Lorenzo Muccioli, Corrado Zenesini, Laura Licchetta, Laura Maria Beatrice Belotti, Lidia Di Vito, Lorenzo Ferri, Domenico Fiorillo, Barbara Mostacci, Elena Pasini, Patrizia Riguzzi, Martina Soldà, Lisa Taruffi, Lilia Volpi, Federico Mason, Francesco Nonino, Roberto Michelucci, Paolo Tinuper, Luca Vignatelli, Francesca Bisulli

**Affiliations:** ^1^ Full Member of the European Reference Network for Rare and Complex Epilepsies (EpiCARE) IRCCS Istituto delle Scienze Neurologiche di Bologna Bologna Italy; ^2^ Department of Biomedical and Neuromotor Sciences University of Bologna Bologna Italy; ^3^ Dipartimento di Ingegneria dell'Informazione DEI University of Padova Padova Italy

**Keywords:** antiseizure medication, cohort studies, drug‐resistant epilepsy, epidemiology, record‐linkage

## Abstract

**Background:**

Epilepsy significantly impacts on morbidity and mortality. Understanding hospitalization and mortality risks in persons with epilepsy (PWE) is essential for improving healthcare strategies. We aimed to investigate the risk and causes of hospitalization and mortality in PWE compared to a matched general population cohort.

**Methods:**

The EpiLink Bologna historical cohort study analyzed adult PWE in the period 2018–2019. A general population control cohort was used for comparison. Clinical data were linked with health administrative data. PWE were grouped into persons with focal epilepsy, idiopathic generalized epilepsy, and developmental and/or epileptic encephalopathy (PDEE). The primary outcome was the hospitalization rate. Emergency department (ED) visit rate and the risk of death for any cause were also assessed.

**Results:**

The study included 1438 PWE and 14,096 controls. PWE had higher incidence rate ratio (IRR) for ED visit (IRR 1.26, 95% CI 1.20–1.32), hospital admission (IRR 2.05, 95% CI 1.83–2.29), and death (IRR 1.5, 95% CI 1.1–2.2) compared to control cohort. The highest hospitalization risk was in the PDEE group (IRR 4.70; 95% CI 3.28–6.74). The increased hospitalization rate among PWE was due to both their higher ED visit and elective hospital admission rates. PWE on polytherapy were at higher risk of hospitalization for inflammation of jaw, acid–base/electrolyte imbalances, chronic cerebrovascular disease, major traumas and infections.

**Conclusions:**

During a 2‐year‐period, PWE in Bologna had a doubled risk of hospitalization and 50% higher risk of death compared to a matched general population cohort. Hospitalization risks varied significantly by epilepsy type and antiseizure therapy.

## INTRODUCTION

Epilepsy is a chronic neurologic condition accounting for a significant proportion of the world's disease burden [1], with a prevalence of almost 1% [2]. People with epilepsy (PWE) are also susceptible to numerous comorbidities and complications, which can arise from common underlying predispositions, epilepsy etiologies, direct effects of seizures, and adverse effects of antiseizure medications (ASM) [3].

Hospital admissions may serve as an indicator of the impact of epilepsy comorbidities, and previous population studies have shown that PWE are more frequently hospitalized [4, 5]. Besides epilepsy and seizures, other common reasons for admissions among PWE include injuries [6], infections [5], vascular disorders [4, 5], and psychiatric conditions [4, 5]. PWE face a higher risk of death than the general population [7]; mortality estimates for epilepsy can vary based on the quality of case ascertainment and other factors [2].

In 2017, the new ILAE classification of the epilepsies was developed [8] and has since been globally implemented [9, 10, 11, 12, 13]. The above reported data on hospital admissions in PWE are based on cohorts that do not utilize the 2017 ILAE classification and do not identify the main categories of epilepsy, such as idiopathic generalized epilepsy, focal epilepsy, developmental and/or epileptic encephalopathy. Furthermore, there is a lack of data on emergency department (ED) visits linked to subsequent hospitalization in PWE. This information is crucial for understanding the healthcare pathway of PWE, distinguishing between urgent and deferrable healthcare requirements, and facilitating the optimization of patient care while rationalizing the utilization of healthcare resources.

Hence, we aimed to investigate the risk and causes of ED visits, hospitalization, and mortality in a large cohort of PWE referred to a tertiary epilepsy center in Bologna, Italy, compared with a matched control population over a 2‐year period, taking into account the main epilepsy types and applying a record‐linkage system, combining both administrative and clinical health data.

## METHOD

### Design

This was a historical cohort study with a matched control cohort. The STROBE (Strengthening the Reporting of Observational Studies in Epidemiology) [14] and the RECORD (The REporting of studies Conducted using Observational Routinely‐collected health Data) [15] guidelines were followed.

### Setting, study population, data sources

The local health trust of Bologna (LHTB), Emilia‐Romagna region, Northern Italy, had a population of approximately 750,000 inhabitants on December 31, 2017.

Patients were recruited at the Epilepsy Center of the “IRCCS Istituto delle Scienze Neurologiche” (Bologna), which follows the vast majority of PWE in the LHTB, ranging from persons referred for a suspected first seizure or a new diagnosis of epilepsy, to persons with rare and complex epilepsies. All adult patients living in the LHTB who underwent a visit during 2016 and 2017, alive on December 31, 2017, were screened by two authors (DF and LM). Those with a diagnosis of epilepsy based on ILAE criteria [16] were included in the study and classified into four categories: idiopathic generalized epilepsy (PIGE, which comprises Childhood Absence Epilepsy, Juvenile Absence Epilepsy, Juvenile Myoclonic Epilepsy, and Generalized Tonic–Clonic Seizures Alone), focal epilepsy (PFE), developmental and/or epileptic encephalopathy (PDEE), and patients with a different or unknown epilepsy type [8] (EpiLink cohort).

The control cohort consisted of people living in the LHTB area, matched to the EpiLink cohort with a ratio of 1:10 for age, sex, district of residence, and comorbidity according to the Charlson comorbidity index [17], based on data from the hospital discharge database.

Each individual of the two cohorts was linked with administrative databases (ED visits, hospital discharges, drug consumption, mortality; almost 100% coverage). A similar approach has been previously used to study COVID‐19 outcomes in PWE and in patients with Parkinson's disease [18, 19].

### Outcomes

The hospital admission rate from January 1, 2018, to December 31, 2019, was the primary outcome. We selected an observation period preceding the onset of the COVID‐19 pandemic to avoid biases related to its consequences on healthcare systems in general and to PWE specifically [20]. Diagnosis was obtained from the hospital discharge administrative database and classified according to the International Classification of Diseases‐9‐CM (ICD‐9‐CM) codes. The ICD‐9‐CM codes (primary level and three secondary levels, Table S1) were grouped into broader categories [21] that explore both the most frequent acute disease conditions and epilepsy‐related chronic diseases. We also assessed the ED visit rate (ICD‐9‐CM unique code) and the risk of death for any cause in both groups during the same period.

### Statistical analysis

Poisson regression models were used to calculate the Incidence Rate Ratio (IRR) with 95% confidence interval (95% CI), for the different outcomes, between the PWE and control cohort. The dependent variable of the models was the count of hospital admissions or ED visits from January 1, 2018, to December 31, 2019; the independent variable was the group classification (PWE vs controls). We also evaluated the IRR in the different diagnostic category PFE, PIGE, and PDEE. The incidence of hospital admission and ED visit for each outcome was calculated as the ratio between the event in the period 2018–2019 and the time‐person in the same time. The conditional probability of hospitalization after an ED visit was calculated comparing the frequencies of hospitalization via ED to the total number of ED visits between PWE and controls.

We performed a subgroup analysis for each outcome stratifying each category of diagnosis into “polytherapy” for persons who were regularly prescribed ≥2 antiseizure medications (ASMs) and “non‐polytherapy” for persons who were prescribed <2 ASMs. For the subgroup analysis, we used multivariable Poisson regression models to estimate the IRR (95% CI) adjusted for sex, age, Charlson comorbidity index, and district of residence. The dependent variable in the multivariable models was the count of hospital admissions or ED visits from January 1, 2018 to December 31, 2019. The independent variable included three categories: PWE on polytherapy versus PWE not on polytherapy versus controls.

Survival analysis was performed to evaluate the mortality between the PWE and control cohort over 2 years of follow‐up. Results were presented as Hazard Ratio (HR) with 95% CI. Stratified analysis was performed by diagnostic category (PFE, PIGE, and PDEE).

Data linkage and statistical analyses were conducted using Stata SE version 14.2.

## RESULTS

**FIGURE 1:**
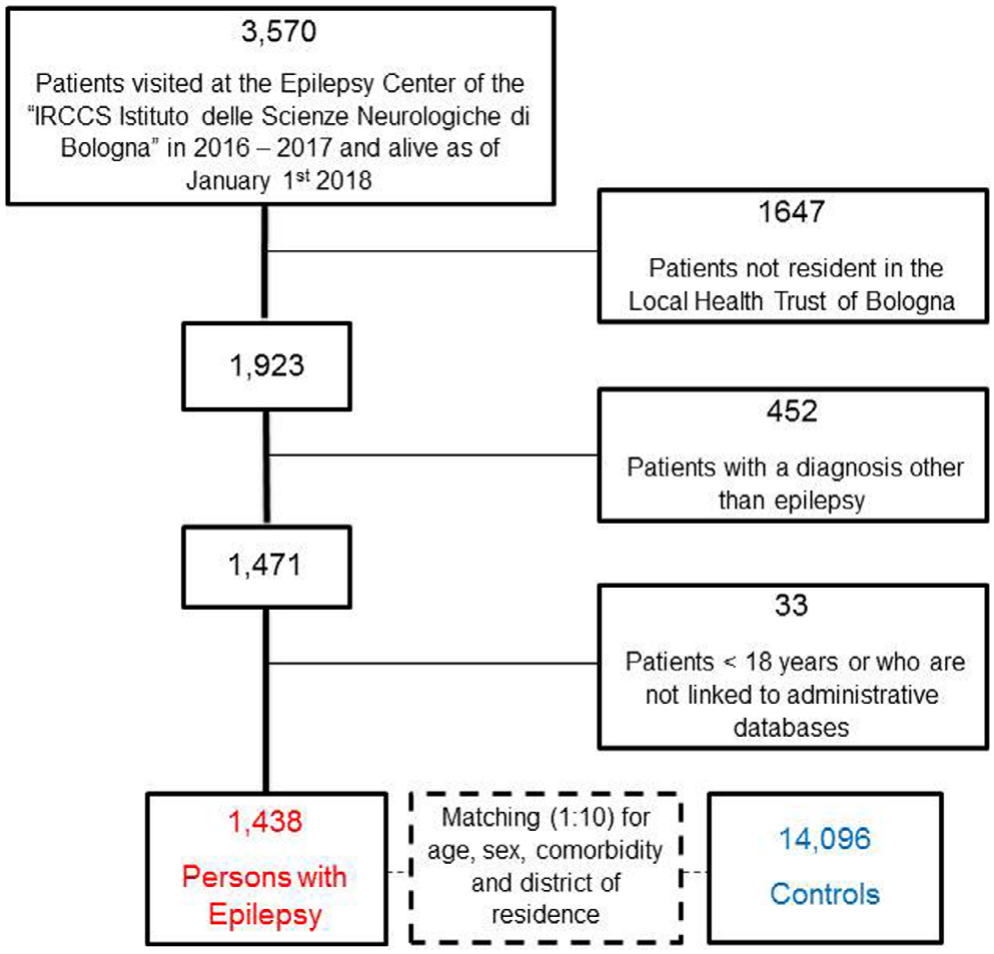
Flowchart illustrating the process of inclusion of people with epilepsy in the EpiLink Bologna cohort and the control cohort.

The EpiLink cohort included 1438 PWE (shown in Figure 1), the majority of which were PFE (*n* = 1025, 71%), followed by PIGE (*n* = 265, 18%), PDEE (*n* = 125, 9%), and patients with a different or unknown epilepsy type (*n* = 23, 2%). The median age was 51 years (IQR 38–65 years), 51% of the cohort was female. The control cohort included 14,096 subjects with the same demographic features (Table 1).

**TABLE 1:** Demographic and main clinical features of the EpiLink cohort and the population‐based matched control cohort.

	Focal epilepsy	Idiopathic generalized epilepsy	Epileptic encephalopathy	*p*‐value^a^	EpiLink cohort^b^	Control cohort
*N*	1025	265	125		1438	14,096
Age, median (IQR)	55 (42–68)	39 (27–53)	40 (26–54)	<0.001	51 (38–65)	51 (38–65)
Sex, *n* (%)						
M	502 (49.0)	123 (46.4)	66 (52.8)	0.197	704 (49.0)	6899 (48.9)
F	523 (51.0)	142 (53.6)	9 (47.2)		734 (51.0)	7197 (51.1)
District, *n* (%)						
Bologna	461 (45.0)	121 (45.7)	66 (52.8)	0.031	653 (45.4)	6440 (45.7)
Reno	129 (12.6)	27 (10.2)	15 (12.0)		176 (12.2)	1728 (12.3)
Pianura Est	190 (18.5)	49 (18.5)	22 (17.6)		269 (18.7)	621 (18.6)
Pianura Ovest	100 (9.8)	28 (10.6)	8 (6.4)		136 (9.5)	1342 (9.5)
Appennino	45 (4.4)	21 (7.9)	6 (4.8)		73 (5.1)	709 (5.0)
San Lazzaro	100 (9.8)	19 (7.2)	8 (6.4)		131 (9.1)	1256 (8.9)
Charlson comorbidity index, *n* (%)						
0	897 (87.5)	250 (94.3)	116 (92.8)	<0.001	1285 (89.4)	12,703 (90.1)
1	48 (4.7)	3 (1.1)	3 (2.4)		54 (3.8)	522 (3.7)
≥2	80 (7.8)	12 (4.6)	6 (4.8)		99 (6.8)	871 (6.2)
Number of antiseizure medications, *n* (%)						
0–1	613 (59.8)	189 (71.3)	55 (44.0)	<0.001	875 (60.8)	‐
≥2	412 (40.2)	76 (28.7)	70 (56.0)		563 (39.2)	
People with^c^, *n* (%)						
At least 1 ED access	509 (49.7)	136 (51.3)	57 (46.3)	0.016	715 (49.8)	4957 (35.2)
At least 1 ED + H access	89 (8.7)	19 (7.2)	6 (4.9)		116 (8.1)	732 (5.2)
At least 1 H access	102 (10.0)	11 (4.2)	16 (13.0)		130 (9.1)	616 (4.4)

^a^ Comparison among the three groups of people with epilepsy.

^b^ Also including 23 patients with unclassified/other epilepsy type.

^c^ ED access = access to emergency department not followed by hospitalization; ED + H access = access to emergency department followed by hospitalization; H access = elective hospitalization.

### ED visits and hospital admissions

Seven hundred and eighty‐nine PWE (55%) had at least one ED visit or hospitalization, compared with 5469 people (39%) in the control cohort.

The incidence of ED visit was 260.9/1000 person‐years (95% CI: 242.8–280.4) in PWE and 192.8/1000 person‐years (95% CI: 187.6–198.1) in controls. The IRR of ED visit in PWE was 1.26 (95% CI: 1.20–1.32) versus the control cohort. The diagnoses observed more commonly in PWE compared to controls included seizure/epilepsy, acid–base and electrolyte imbalances, acute cerebrovascular events, major traumas, hypotension, and infections (Table 2).

**TABLE 2:** Incidence rate for the different outcomes of emergency department visit, and corresponding Incidence Rate IRR with 95% CI between the EpiLink and control cohorts.

	EpiLink cohort incidence	Control cohort incidence	IRR (95% CI)	*p*
	(n/1000‐person‐year)			
Any Emergency Department access	260.9	192.8	1.26 (1.20–1.32)	<0.001
Seizure/epilepsy	54.5	0.4	188 (105–336)	<0.001
Major traumas	45.0	19.1	2.54 (2.14–3.02)	<0.001
Infections	12.3	8.6	1.60 (1.16–2.21)	0.004
Hypotension	7.7	5.0	1.62 (1.05–2.49)	0.030
Acute cerebrovascular event	4.2	1.7	2.78 (1.54–5.05)	0.001
Headache	3.9	3.2	1.22 (0.68–2.17)	0.503
Hypertensive disease	3.9	3.0	1.21 (0.65–2.26)	0.554
Acid–base/electrolyte imbalances	3.2	0.4	8.76 (3.87–19.9)	<0.001

*Note*: Events are those with at least 3/1000‐person‐year of incidence and reported according to the rank of incidence in the EpiLink cohort.

The incidence of hospitalization (both elective and after ED visit) was 78.8/1000 person‐years (95% CI: 69.1–89.8) in PWE and 44.3/1000 person‐years (95% CI: 41.9–46.9) in controls. The IRR of hospitalization in PWE was 2.05 (95% CI: 1.83–2.29) compared to the control cohort. The IRR was 1.72 (95% CI: 1.46–2.02) for hospitalization via ED, and 2.43 (95% CI: 2.09–2.83) for elective hospitalization. The conditional probability of hospital admission after an ED visit was 9.3% in PWE and 9.4% in controls (*p* = 0.98).

The most frequent diagnoses for PWE via ED visit were seizure/epilepsy, chronic cerebrovascular disease, acid–base/electrolyte imbalances, dementia, infections, and major traumas (Table 3). The most frequent diagnoses for PWE for elective hospitalization were seizure/epilepsy, cerebral tumors, inflammation of jaw, anemias, infections, and chronic cerebrovascular disease (Table 3).

**TABLE 3:** Incidence rate for the different hospital clinical features (displayed also for admissions following ED access, and elective hospital admissions), and IRR with 95% CI between the EpiLink and control cohorts.

	EpiLink cohort incidence	Control cohort incidence	IRR (95% CI)	*p*
	(n/1000‐person‐year)			
Any hospital admission	78.8	44.3	2.05 (1.83–2.29)	<0.001
Via ED access	41.8	27.3	1.72 (1.46–2.02)	<0.001
Elective admission	46.8	23.1	2.43 (2.09–2.83)	<0.001
Seizure/epilepsy	29.5	0.5	75.8 (44.3–123)	<0.001
Via ED access	19.7	0.4	61.7 (32.7–116)	<0.001
Elective admission	12.3	0.07	115 (41.4–318)	<0.001
Chronic cerebrovascular disease	9.1	1.9	4.21 (2.74–6.48)	<0.001
Via ED access	6.4	1.2	5.57 (3.25–9.55)	<0.001
Elective admission	3.2	0.8	2.69 (1.28–5.63)	0.009
Infections	8.4	4.5	2.61 (1.83–3.71)	<0.001
Via ED access	7.7	4.6	2.37 (1.63–3.43)	<0.001
Elective admission	2.5	0.6	4.53 (2.05–10.0)	<0.001
Major traumas	7.7	3.9	2.09 (1.37–3.19)	0.001
Via ED access	7.0	3.3	2.18 (1.36–3.50)	0.001
Elective admission	1.4	0.9	1.77 (0.68–4.60)	0.241
Hypertensive disease	7.0	6.8	0.92 (0.59–1.45)	0.733
Via ED access	5.3	5.2	0.92 (0.54–1.57)	0.773
Elective admission	2.1	2.0	0.92 (0.40–2.14)	0.855
Ischemic heart disease	6.0	4.5	1.34 (0.87–2.09)	0.179
Via ED access	4.2	3.2	1.15 (0.65–2.05)	0.633
Elective admission	3.2	1.9	1.74 (0.89–3.41)	0.108
Inflammation of jaw	5.2	0.5	14.3 (7.30–28.2)	<0.001
Via ED access	0	0	n.c.	n.c.
Elective admission	5.3	0.5	14.3 (7.30–28.2)	<0.001
Acute cerebrovascular event	4.6	3.1	1.40 (0.83–2.37)	0.206
Via ED access	3.9	2.5	1.75 (0.97–3.16)	0.064
Elective admission	1.0	1.1	0.75 (0.23–2.44)	0.639
Acid–base/electrolyte imbalances	4.6	1.0	2.64 (1.41–4.90)	0.002
Via ED access	4.2	0.9	5.35 (2.78–10.3)	<0.001
Elective admission	0.4	0.2	1.91 (0.22–16.4)	0.554
Cardiac dysrhythmia/Conduction heart disorder	3.9	4.9	1.22 (0.78–1.93)	0.384
Via ED access	2.8	3.4	1.33 (0.77–2.28)	0.304
Elective admission	1.4	1.8	1.02 (0.44–2.38)	0.956
Anemias	3.5	2.1	2.12 (1.19–3.79)	0.011
Via ED access	2.5	2.0	1.37 (0.65–2.86)	0.410
Elective admission	1.8	0.3	8.19 (2.75–24.4)	<0.001
Diabetes	3.5	4.3	0.92 (0.55–1.53)	0.736
Via ED access	2.8	3.2	0.85 (0.45–1.63)	0.631
Elective admission	1.4	1.8	1.04 (0.45–2.42)	0.923
Liver diseases	3.2	2.1	1.48 (0.78–2.79)	0.226
Via ED access	1.4	1.4	1.11 (0.44–2.81)	0.823
Elective admission	2.0	1.0	2.05 (0.85–4.95)	0.111
Dementia	3.2	1.1	3.39 (1.70–6.75)	0.001
Via ED access	3.2	0.9	4.04 (2.00–8.18)	<0.001
Elective admission	0.0	0.2	n.c.	n.c.
Cerebral tumor	3.2	0.3	17.9 (9.80–32.9)	<0.001
Via ED access	0.3	0.1	3.18 (0.33–30.6)	0.316
Elective admission	3.2	0.2	21.3 (11.1–41.0)	<0.001

*Note*: Events are those with at least 3/1000‐person‐year of incidence and reported according to the rank of incidence in the EpiLink cohort.

A more detailed analysis of the causes of hospitalization revealed that infections affected 12 PWE (15 events, 6 in PDEE), with respiratory infections being the most frequent cause (all 12 PWE, 14 hospitalizations). Major traumas affected 18 PWE (22 events). Skull traumas were the most frequent event (10), followed by lower limbs (6), upper limbs (3), pelvic bones (3), and chest (2) traumas/fractures. Twelve PWE were hospitalized for inflammation of the jaw, an ICD label that applies to abscess, osteitis, periostitis, or sequestrum of the jaw, which was associated with elective tooth intervention (removal of impacted tooth or residual root, 8; restoration of tooth by filling, 5; prosthetic dental implant, 3; and other dental operations, 1), mouth/facial surgery (local excision or destruction of lesion of facial bone, 4; excision of dental lesion of jaw, 1; and suture of laceration of other part of mouth, 1), or invasive diagnostic procedures on teeth, gums, and alveoli (8). Only one out of 12 PWE was treated with phenytoin.

### Categories of epilepsy

The subgroup at highest risk of accessing ED was PDEE (IRR: 1.56, 95% CI: 1.31–1.85); PFE and PIGE had IRRs of 1.24 (95% CI: 1.17–1.32) and 1.23 (95% CI: 1.09–1.38), respectively. The frequency of hospital admission after an ED visit differed only in the PDEE diagnostic category (13.5% vs 5.5% in controls, *p* < 0.001).

The subgroup with the highest risk of hospitalization was PDEE (IRR: 4.70, 95% CI: 3.28–6.74); PFE and PIGE had IRRs of 1.94 (95% CI: 1.70–2.20) and 1.75 (95% CI: 1.31–2.35), respectively. Regarding discharge codes by subgroups (Table 4), seizure/epilepsy, chronic cerebrovascular disease, major traumas, and infections were found to be consistently more frequent in all subgroups. Dementia and acid–base and electrolyte imbalances resulted at higher risk in PFE and PIGE, jaw inflammation emerged as more frequent in PFE and PDEE, while cerebral tumors and ischemic heart disease only in PFE and acute cerebrovascular events in PDEE.

**TABLE 4:** Incidence Rate Ratio (IRR) with 95% 95% CI for the different Hospital clinical features in the three categories of epilepsy (focal epilepsy, idiopathic generalized epilepsy, epileptic encephalopathy) versus matched control cohorts.

	Focal epilepsy	Idiopathic generalized epilepsy	Epileptic encephalopathy
	IRR (95% CI)	IRR (95% CI)	IRR (95% CI)
Any hospital admission	1.94 (1.70–2.20)	1.75 (1.31–2.35)	4.70 (3.28–6.74)
Seizure/epilepsy	90.5 (45.5–180)	20.8 (7.22–59.9)	211 (28.5–1568)
Cerebral tumor	19.2 (10.3–35.6)	n.c.	n.c.
Inflammation of jaw	9.59 (4.44–20.7)	18.9 (1.71–209)	n.c.^a^
Chronic cerebrovascular disease	3.36 (2.02–5.60)	3.78 (1.19–12.1)	57.7 (6.94–479)
Dementia	2.74 (1.25–6.01)	14.2 (2.37–84.7)	n.c.
Acid–base/electrolyte imbalances	2.68 (1.16–6.21)	4.73 (0.87–25.8)	n.c.
Anemias	2.25 (1.20–4.23)	n.c.	6.40 (1.07–38.3)
Infections	1.86 (1.23–2.18)	5.0 (2.31–10.7)	33.6 (6.99–162)
Major traumas	1.72 (1.02–2.87)	5.0 (2.14–11.9)	1.92 (0.22–16.5)
Ischemic heart disease	1.67 (1.06–2.63)	n.c.	3.20 (0.33–30.8)
Acute cerebrovascular event	1.25 (0.69–2.28)	1.26 (0.29–5.51)	9.61 (1.35–68.2)

*Note*: Events are reported according to the rank of IRR in the focal epilepsy cohort.

Abbreviation: n.c, not calculable for absence of events.

^a^ Six cases in people with people epileptic encephalopathy and none in the control group.

At multivariable analysis, PWE on polytherapy compared to those on monotherapy showed a higher risk of ED visit for any cause, seizure/epilepsy, acid–base/electrolyte imbalances, and major traumas. Similarly, the polytherapy category was at higher risk of hospitalization for any admission, seizure/epilepsy, inflammation of jaw, acid–base/electrolyte imbalances, chronic cerebrovascular disease, major traumas and infections (Table 5).

**TABLE 5:** Role of ASM treatment (monotherapy and polytherapy subgroups) in EpiLink cohort versus control cohort. IRR with 95% CI are reported for the different outcomes of emergency department and hospital admissions according to the rank of IRR in the EpiLink cohort and for the significant outcomes at the main analysis (see Tables 2 and 3), adjusted for covariates^a^ (multivariable Poisson regression models).

	EpiLink cohort versus control cohort: Emergency department admissions		EpiLink cohort versus control cohort Hospital admissions	
	IRR (95% CI)	*p*	IRR (95% CI)	*p*
Any admission				
Monotherapy	1.21 (1.13–1.29)	0.020	1.77 (1.53–2.06)	0.009
Polytherapy	1.35 (1.26–1.45)		2.32 (1.99–2.72)	
Seizure/epilepsy				
Monotherapy	156 (83.8–289)	<0.001	46.3 (24.6–86.8)	<0.001
Polytherapy	251 (135–466)		130 (72.2–235)	
Cerebral tumor				
Monotherapy	–		17.1 (8.8–33.1)	0.499
Polytherapy			13.1 (5.9–29.1)	
Inflammation of jaw				
Monotherapy	–		6.61 (2.52–17.1)	0.008
Polytherapy			23.8 (11.4–49.4)	
Acid–base/electrolyte imbalances				
Monotherapy	5.78 (1.84–18.19)	0.132	2.30 (0.80–6.58)	0.018
Polytherapy	14.9 (5.76–38.6)		9.17 (4.53–18.5)	
Chronic cerebrovascular disease				
Monotherapy	–		2.57 (1.32–5.01)	0.014
Polytherapy			6.74 (4.02–11.3)	
Infections				
Monotherapy	1.23 (0.79–1.93)	0.065	1.84 (1.12–3.00)	0.014
Polytherapy	2.15 (1.41–3.26)		3.95 (2.59–6.04)	
Major traumas				
Monotherapy	1.85 (1.45–2.36)	<0.001	0.92 (0.43–1.98)	0.002
Polytherapy	3.57 (2.86–4.45)		3.70 (2.25–6.08)	
Anemias				
Monotherapy	–		1.23 (0.50–3.08)	0.115
Polytherapy			3.04 (1.45–6.37)	
Dementia				
Monotherapy	–		2.84 (1.17–6.88)	0.507
Polytherapy			1.65 (0.39–6.91)	

*Note*: *p*‐values correspond to statistical difference between monotherapy and polytherapy.

^a^ Age, sex, Charlson index, district of residence.

### Mortality

At 2 years, mortality for any cause was 2.6% in PWE and 1.8% in controls (*p* = 0.015), corresponding to an hazard ratio of death of 1.5 (95% CI: 1.1–2.2), with an higher risk for PFE (1.5, 95% CI: 1.01–2.1) and PDEE (13.8, 95% CI: 3.1–61.7), but not in PIGE (0.72, 95% CI: 0.17–3.01).

## DISCUSSION

During a 2‐year period, PWE in the local health trust of Bologna had 25% higher rate of ED visits and a twofold rate of hospital admissions compared with a matched general population cohort. Besides seizures and epilepsy being expected leading causes of hospitalization, further potential causes of admissions revealed two patterns of higher risks in PWE. Firstly, as direct or indirect consequences of epilepsy, PWE were frequently hospitalized for infections, major traumas, jaw/dental disorders, acid–base/electrolyte imbalances, and anemias. Secondly, hospitalization for cerebrovascular disease, dementia, and cerebral tumors reflected well‐known causes or comorbidity of epilepsy [22, 23, 24]. PWE were confirmed to be at higher mortality risk compared to a control cohort [2, 25, 26]. A focus on causes of death was beyond the scope of this study; however, among those reported in the literature [26], brain tumors, pneumonia, injuries, cerebrovascular disease were the leading causes of ED access or hospitalization in our cohort.

The risk of hospitalization varied according to the type of epilepsy: PFE was the largest group and drove the overall IRR, while PDEE had the highest risk, that is, almost five times higher than the control population. This finding reflects the fact that, among PWE, PDEE represent the most vulnerable patients, are frequently institutionalized, and might have a higher risk of developing infections and other complications, in agreement with previous reports [27]. The polytherapy, probably in people with drug‐resistant epilepsy [5, 28], arguably contributed to the “additive” predisposition for jaw/dental disorders, acid–base and electrolyte imbalances, infections, and major traumas.

The increased hospitalization rate among PWE was due to both their higher ED and elective hospital admission rates compared to controls, while the likelihood of hospital admission following an ED visit was notably higher only for PDEE and not for PWE in general, implying that the decision‐making process in the ED is likely influenced by more severe epilepsy phenotypes.

PWE were at increased risk of developing infections, particularly respiratory infections, compared to the general population. Septicemia and pneumonia were reported among the most frequent causes of nonelective hospitalizations in PWE in a US population‐based study [5]. This risk was particularly higher in PDEE, possibly due to their comorbidities and the frequent institutionalization [27]. However, biological factors may also be involved, such as the low vitamin D levels reported to be common in PWE on ASM [29]. Vaccination campaigns (e.g., against influenza, COVID‐19, and pneumococcal infections) should be encouraged to reduce the risk of severe infections and their complications.

Major traumas, particularly skull and lower limb fractures, were more frequently causes of hospitalization in PWE. Admission via ED access testifies to an acute event likely related to seizures, as these may lead to loss of consciousness, loss of muscle control, and other symptoms that can make it difficult to maintain balance and avoid obstacles [30]. The risk is particularly high in PWE with polytherapy reflecting both a refractory epilepsy with traumatic seizures, and an impairment of balance or impairment of postural reflexes as adverse effect of ASMs. An actionable measure that can be proposed to mitigate this risk in the population with frequent traumatic seizures includes promoting the use of protective devices, such as helmets.

A remarkable cause of elective hospitalization in PWE was jaw/dental disorders leading to dental interventions or mouth/facial surgery. This finding may be attributed to the higher risk of oral and maxillofacial injuries in PWE than healthy individuals [31], as well as a higher likelihood of poor oral hygiene, gingivitis, and periodontitis [32, 33], especially in PDEE. Therefore, regular dental check‐ups should be encouraged to prevent dental issues in this population.

PWE may be predisposed to developing electrolyte and acid–base balance disturbances, especially if they are taking certain type of ASM. Sodium‐channel blockers such as carbamazepine and oxcarbazepine can cause hyponatremia in approximately 13% and 30% of treated patients, respectively, which might be severe in a significant proportion of cases and lead to hospital admission [34]. Other ASM have the potential to cause metabolic acidosis: valproic acid through the development of hyperammonemia, topiramate and zonisamide by inhibiting the activity of carbonic anhydrase, leading to renal loss of bicarbonate [35].

Anemia, another condition that occurred more frequently in elective hospital admissions of PWE, represents a possible adverse events of various ASMs, either due to direct cytotoxic effects (e.g., aplastic anemia due to carbamazepine, phenytoin, or valproate), immunologic reactions (e.g., hemolytic anemia with carbamazepine), or cumulative dose effects (e.g., megaloblastic anemia due to folate deficiency associated with long‐term use of carbamazepine or valproate) [36].

As ASM may contribute to several of the more frequently observed diagnoses in PWE, optimizing treatment regimens is crucial. This can be achieved by rationalizing polytherapy through the use of safer drug combinations that leverage different mechanisms of action [37]. Tailoring treatment in this way not only minimizes potential side effects but also reduces the risk of drug–drug interactions, adverse events, and long‐term complications associated with certain ASMs [38].

This study has certain limitations, Firstly, detailed information on the severity of epilepsy was not available; however, the number of ASM used can be considered as a proxy for disease severity. Consistently, the multivariable analysis revealed a higher risk of several outcomes in the polytherapy group. Secondly, administrative data were used for outcome assessment, which may have led to underestimation or overestimation of certain outcomes. However, the same distortion would affect the control cohort as well, minimizing the bias of the IRRs. Lastly, our cohort was built recruiting patients from a tertiary level center. However, our institute follows both persons with a new diagnosis of epilepsy and those with rare and complex epilepsies; therefore, our sample is representative of a wide spectrum of PWE.

In conclusion, our study adds new insights into the modality and causes of increased access to ED visits and elective hospitalizations among different etiologic subgroups of PWE, with important implications for both clinical practice and public health. It is crucial to pay special attention to more vulnerable subjects (PDEE) and to environmental safety factors that contribute to ED visits, such as addressing trauma and infectious risks, as well as adverse events of ASMs in PWE on polytherapy using safer combinations of drugs. Additionally, PWE often have chronic comorbidities (such as jaw/dental disorders, chronic cerebrovascular disease, dementia, and cerebral tumors) that contribute to elective hospitalizations. Further research is needed to identify effective interventions that can reduce epilepsy‐related risks.

## AUTHOR CONTRIBUTIONS

**Lorenzo Muccioli:** Conceptualization; investigation; writing – original draft; data curation. **Corrado Zenesini:** Conceptualization; methodology; data curation; formal analysis; writing – original draft. **Laura Licchetta:** Investigation; writing – review and editing. **Laura Maria Beatrice Belotti:** Data curation; formal analysis; writing – review and editing. **Lidia Di Vito:** Investigation; writing – review and editing. **Lorenzo Ferri:** Investigation; writing – review and editing. **Domenico Fiorillo:** Data curation; writing – review and editing. **Barbara Mostacci:** Investigation; writing – review and editing. **Elena Pasini:** Investigation; writing – review and editing. **Patrizia Riguzzi:** Investigation; writing – review and editing. **Martina Soldà:** Data curation; writing – review and editing. **Lisa Taruffi:** Data curation; writing – review and editing. **Lilia Volpi:** Investigation; writing – review and editing. **Federico Mason:** Data curation; writing – review and editing. **Francesco Nonino:** Writing – review and editing; methodology. **Roberto Michelucci:** Investigation; writing – review and editing. **Paolo Tinuper:** Supervision; investigation; writing – review and editing. **Luca Vignatelli:** Conceptualization; methodology; formal analysis; writing – original draft. **Francesca Bisulli:** Conceptualization; investigation; writing – original draft; supervision; project administration.

## FUNDING INFORMATION

The study received no external funding.

## CONFLICT OF INTEREST STATEMENT

None of the authors have conflict of interest to disclose.

## ETHICS STATEMENT

This study protocol was reviewed and approved by the local institutional review board (Comitato Etico Area Vasta Emilia Centro; reference number: 574‐2021‐OSS‐AUSLBO).

## CONSENT TO PARTICIPATE STATEMENT

Taking into account the retrospective nature of the study, whenever possible, patients were asked for written consent for the use of personal data. If consent could not be obtained from some patients, considering that failure to include all cases would result in selection bias, provision was made for not acquiring specific consent, according to the rules of the institutional review board. The persons comprising the control cohort are anonymous.

## Supporting information


Table S1.

## Data Availability

The study data are available upon reasonable request.
