# Supplementary material for: Causes of hospitalization and mortality in persons with epilepsy: The EpiLink Bologna cohort, Italy
Source: Eur J Neurol. 2025 Jan 31;32(2):e16576. doi: 10.1111/ene.16576 (PMC11783224; doi:10.1111/ene.16576)
Supplement: Supplementary file 1 — Table S1. [file ENE-32-e16576-s001.docx]

**Causes of hospitalization and mortality in persons with epilepsy:
the EpiLink Bologna cohort, Italy.**Muccioli et al. v 2024-07-12

TABLE S1: Outcome measures and corresponding ICD-9-CM codes of identification.

| **Outcome measurement** | **ICD-9-CM codes** |
| --- | --- |
| **General** |  |
| Any hospitalization | Any hospitalization recorded in the administrative database of hospital discharges |
| **Specific** |  |
| Hospitalizations/ED access for malignant neoplasm of brain | 191 Malignant neoplasm of brain |
| Hospitalizations/ED access for disorders of thyroid gland | 240-246 Disorders of thyroid gland |
| Hospitalizations/ED access for diabetes mellitus | 250 Diabetes mellitus |
| Hospitalizations/ED access for metabolic imbalances | 275 Disorders of mineral metabolism  276 Disorders of fluid, electrolyte and acid-base balance. |
| Hospitalizations/ED access for anemias | 280-285 Anemias |
| Hospitalizations/ED access for dementias | 290 Dementias |
| Hospitalizations/ED access for condition alcohol or drug related | 291 Alcohol-induced mental disorders  292 Drug-induced mental disorders  303 – 305 Alcohol dependence syndrome/ Drug dependence/Nondependent abuse of drugs |
| Hospitalizations/ED access for psychiatric events | 293 Transient mental disorders due to conditions classified elsewhere  295 Schizophrenic disorders  296.0-296.3 [Bipolar I disorder, single manic episode](javascript:void();); [Manic disorder recurrent episode](javascript:void();); [Major depressive disorder single episode](javascript:void();); [Major depressive disorder recurrent episode](javascript:void();)  296.4-296.8 [Bipolar I disorder, most recent episode (or current) manic](javascript:void();); [Bipolar I disorder, most recent episode (or current) depressed](javascript:void();); [Bipolar I disorder, most recent episode (or current) mixed](javascript:void();); Bipolar i disorder, most recent episode (or current) unspecified; [Other and unspecified bipolar disorders](javascript:void();)  296.9 [Other and unspecified episodic mood disorder](javascript:void();)  297-299 [Delusional disorders](javascript:void();), [Other nonorganic psychoses](javascript:void();), [Pervasive developmental disorders](javascript:void();)  298.0 Depressive type psychosis  300.4 Dysthymic disorder  309.0 Adjustment disorder with depressed mood  309.1 Adjustment reaction with prolonged depressive reaction  311 [Depressive disorder not elsewhere classified](javascript:void();) |
| Hospitalizations/ED access for disorders of sleep | 307.4, 327, 780.5 disorders of sleep |
| Hospitalizations/ED access for mental retardation | 317-319 Mental retardation |
| Hospitalizations/ED access for headache / migraine | 307.81 Tension headache  346 Migraine  784.0 Headache |
| Hospitalizations/ED access for epilepsy | 345 Epilepsy and recurrent seizures  649.4 Epilepsy complicating pregnancy, childbirth or puerperium  780.3 Convulsions |
| Hospitalizations/ED access for hypertensive disease | 401-405 Hypertensive disease |
| Hospitalizations/ED access for ischemic heart disease | 410-414 Ischemic heart disease |
| Hospitalizations/ED access for Conduction disorders/Cardiac dysrhythmias | 426 Conduction disorders  427 Cardiac dysrhythmias |
| Hospitalizations/ED access for Heart failure | 428 Heart failure (cardiac decompensation) |
| Hospitalizations/ED access for acute cerebrovascular events | 430-434, 436 Cerebrovascular diseases (excluding TIA, other and poorly defined cerebrovascular diseases, sequelae) |
| Hospitalizations/ED access for chronic cerebrovascular disease | 437 Other and ill-defined cerebrovascular disease  438 Late effects of cerebrovascular disease |
| Hospitalizations/ED access for liver/biliary tract disease | 570-576 for liver/biliary tract disease |
| Hospitalizations/ED access for renal disease | 580-589 nephritis, nephrotic syndrome, and nephrosis  590 Infections of kidney  592 Calculus of kidney and ureter  593 Other disorders of kidney and ureter |
| Hospitalizations/ED access for thromboembolic events | 451.0-451.2 Phlebitis and thrombophlebitis of lower extremities  453.4 Acute venous embolism and thrombosis of unspecified deep vessels of lower extremity  415.1 Pulmonary embolism and infarction |
| Hospitalizations/ED access for hypotension / syncope | 458 Hypotension  780.2 Syncope and collapse |
| Hospitalizations/ED access for infections | 038 Septicemia  480-486 Pneumonia  487.0 With pneumonia  466 Acute bronchitis and bronchiolitis,  507 Pneumonia due to solids and liquids  595 Cystitis  599.0 Urinary tract infection, site not specified  707.0 Pressure ulcer  785.52 Septic shock  995.91 Sepsis  995.92 Severe sepsis |
| Hospitalizations/ED access for diseases of intestines and peritoneum | 560-569 other diseases of intestines and peritoneum  578 Gastrointestinal hemorrhage |
| Hospitalizations/ED access for diseases of the jaws | 526 Diseases of the jaws |
| Hospitalizations/ED access for major traumas | 800-829 Fractures of the skull / neck and trunk / upper and lower limbs  850-854 Intracranial injuries, excluding those associated with skull fractures  717 Internal knee injuries  920 Contusion of the face, scalp and neck excluding the eye  905.2-905.4 Sequelae of fractures of the upper limbs / femoral neck / lower limbs  733.13 Pathological fracture of the vertebrae  922.31 Contusion of the back  924.01 Contusion of the hip  959.11-959.12 Other types of crushing of the chest wall /abdomen  959.19 Other types of chest trauma |
